# Investigation of Cytotoxic T Lymphocyte Function during Allorejection in the Anterior Chamber of the Eye

**DOI:** 10.3390/ijms21134660

**Published:** 2020-06-30

**Authors:** Hsin-Fang Chang, Marie-Louise Wirkner, Elmar Krause, Jens Rettig

**Affiliations:** Cellular Neurophysiology, Center for Integrative Physiology and Molecular Medicine (CIPMM), Saarland University, 66421 Homburg, Germany; fine8mai@gmail.com (M.-L.W.); elmar.krause@uks.eu (E.K.)

**Keywords:** cytotoxic T lymphocyte(s), anterior chamber of the eye, allorejection, islet(s) of Langerhans, two-photon microscopy

## Abstract

Cytotoxic T lymphocytes (CTL) are an essential part of our immune system by killing infected and malignant cells. To fully understand this process, it is necessary to study CTL function in the physiological setting of a living organism to account for their interplay with other immune cells like CD4^+^ T helper cells and macrophages. The anterior chamber of the eye (ACE), originally developed for diabetes research, is ideally suited for non-invasive and longitudinal in vivo imaging. We take advantage of the ACE window to observe immune responses, particularly allorejection of islets of Langerhans cells by CTLs. We follow the onset of the rejection after vascularization on islets until the end of the rejection process for about a month by repetitive two-photon microscopy. We find that CTLs show reduced migration on allogeneic islets in vivo compared to in vitro data, indicating CTL activation. Interestingly, the temporal infiltration pattern of T cells during rejection is precisely regulated, showing enrichment of CD4^+^ T helper cells on the islets before arrival of CD8^+^ CTLs. The adaptation of the ACE to immune responses enables the examination of the mechanism and regulation of CTL-mediated killing in vivo and to further investigate the killing in gene-deficient mice that resemble severe human immune diseases.

## 1. Introduction

Cytotoxic T lymphocytes (CTLs) play an important role in the adaptive immune system. They fight against tumor and virus-infected cells through delivery of cytotoxic components to target cells, thereby inducing apoptotic death. The killing process initiates from target cell recognition through T cell receptor (TCR) engagement with major histocompatibility class I complexes (MHC I) present on target cells. TCR engagement then leads to T cell activation and this engagement triggers the accumulation of signaling molecules at the contact site between CTL and target cell, the immunological synapse (IS). CTL activation through TCR leads to an increase in intracellular calcium and the polarization of cytotoxic granules (CG) towards the IS. CTLs finally fulfill their killing task by releasing cytotoxic substances like granzyme B (GzmB) and perforin from CG onto the target cell. Due to their cytotoxicity, CTLs also play an important role in allograft rejection, mainly to destroy the foreign tissue to maintain the physiological integrity of the organism.

The immune response against transplanted allografts is one of the most effective processes performed by the immune system. The acute rejection response has been attributed to donor dendritic cells (DCs), which migrate from the graft to secondary lymphoid tissue presenting donor MHC molecules and directly prime host T cells, resulting in alloreactive T cell activation (direct pathway). As DCs express both MHC class I and class II, donor antigen can be presented to either CD4^+^ or CD8^+^ T cells and trigger their activation. Apart from direct alloantigen presentation from host DCs, alloreactive T cells can also be activated by recipient antigen-presenting cells (APC), which present allopeptide–self MHC complexes on their surface (indirect pathway) [1]. In addition, alloreactive T cells can also be stimulated through recognition of allogeneic MHC molecules present on recipient APCs (MHC cross-dressing) after their transfer via cell–cell contact or through extracellular vesicles (semi-direct pathway) [2,3,4]. The recipient DCs that acquire exosomes secreted by donor DCs are being activated and present donor MHC to trigger full activation of alloreactive T cells [5]. Moreover, it has been shown that the purified allogeneic exosomes induced proinflammatory alloimmune responses by T cells [6].

Given that alloreactive T cells play an important role in allograft immunity, damage of the allograft tissue is frequently mediated by activated cytotoxic cells. The clearance of the allograft involves several cell populations which may mediate cellular cytotoxicity, including allospecific CD3^+^CD8^+^ CTLs, CD3^+^CD4^+^ T helper cells and natural killer cells which do not require MHC allorecognition to induce cytotoxicity. It has been shown that early onset of cellular rejection correlates with an increase in GzmB expression [7,8,9].

With regard to the importance of allorejection for the human immune system, it is important to investigate its individual components with high temporal and spatial resolution in an intact organism in order to understand the molecular mechanism. The recently established anterior chamber of the eye (ACE) technology is ideally suited to study CTL effector function in vivo. The ACE was initially described as a novel imaging site in diabetes research by observing transplanted pancreatic islet activity in the eye of a living animal [10,11]. The ACE technology is ideally suited for non-invasive and longitudinal in vivo imaging [12]. We take advantage of the ACE window to observe immune responses, particularly allorejection of islet cells by CTLs. We transplanted islets of Langerhans from DBA/2J mice into a C57BL/6 recipient mice line to generate an allograft rejection.

We strategically use different recipient mice that either label all various T cell populations (Bonzo mouse; [13]), label only CD8^+^ T cells (CD8-τGFP; see Section 4.1) or label only CG within CTLs (GzmB KI and SybKI; see Section 4.1) to investigate the effector function of CTLs against allograft islet with cellular or subcellular resolution. We follow the onset of the rejection after vascularization on islets, when immune cells start to infiltrate into ACE, until the end of the rejection process, where the islets are entirely removed for about a month by repetitive two-photon microscopy. Additionally, we observe the different infiltration kinetics of alloreactive CD4^+^ helper T cells and CD8^+^ CTLs against the allografts by histological analysis. We demonstrate that the ACE platform is ideally suited to study the CTL-mediated removal of allograft islets with single-cell resolution in vitro and in vivo over extended time periods.

## 2. Results

### 2.1. Visualization of Infiltrated Cytotoxic T Lymphocytes and Their Effector Function on Pancreatic Islets In Vitro

**Figure 1:**
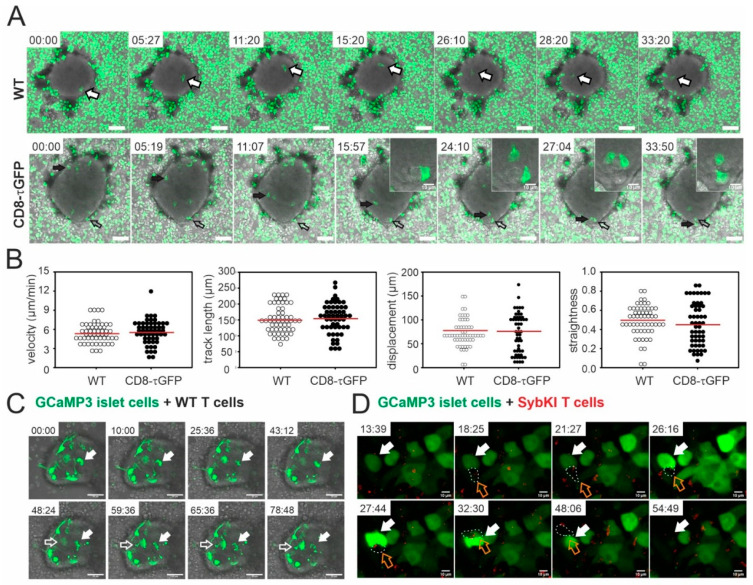
Confocal live imaging of CTLs infiltrating and killing pancreatic islet cells in vitro. (**A**) Confocal snapshots from live images of calcein-loaded WT and CD8-*τ*GFP CTLs (green) infiltrating an islet. Arrows point to individual CTLs. Scale bar: 50 µm. Inset shows a CTL (open arrow in overview) in contact with an islet cell. The images were acquired in 9 µm slices with 1 µm interval distance and ~0.3 Hz acquisition frequency. The entire recording is shown in Movies S1 and S2. (**B**) Quantitative analysis of velocity, track length, displacement and straightness of WT and CD8-*τ*GFP CTLs on DBA/2-derived islets of Langerhans. (**C**) Confocal snapshots from live images of WT CTLs co-cultured with islet cells which were infected with AV-GCaMP3 (green). Arrows point to apoptotic islet cells whose death can be followed in Movie S3. Scale bar: 50 µm. The images were acquired in 12 µm slices with 2 µm interval distance and ~0.2 Hz acquisition frequency. (**D**) Confocal snapshots from live images of SybKI CTLs (red) co-cultured with islet cells which were infected with AV-GCaMP3 (green). The white arrows point to an islet cell that showed a rapid increase in intracellular calcium concentration after contact with a single CTL (orange arrows). Scale bar: 50 µm. The images were acquired in 5 µm slices with 1 µm interval distance and ~0.1 Hz acquisition frequency. Maximum intensity projections are shown.

As powerful as the ACE model is (see Section 2.2, Section 2.3 and Section 2.4), it requires sophisticated imaging techniques over an extended time period. Therefore, we first wanted to prove that T cells can efficiently kill islets of Langerhans from allogenic mice in vitro. For this purpose, we generated T cells that specifically recognize DBA/2 antigens by co-culturing splenocytes from C57BL/6 mice with DBA/2 exocrine material from islet preparations (see Section 4.4) for two days. We initially observed blast T cells in this mixed culture, and after addition of 100 U/mL interleukin-2 (IL-2), the T cells started to proliferate. We purified T cells from this culture and verified that cultured cells from three different mouse lines that contained CD8^+^ CTLs as the major population (Figure S1). We distinguished CD4^+^ T helper cells from CTLs by labeling with anti-CD4 and anti-CD8 antibodies, respectively. After five days, we found 77.5% CD8^+^ and 5.1% CD4^+^ cells in cultures from wild-type (WT) mice, 93.5% CD8^+^ and 6.5% CD4^+^ cells in cultures from GzmB KI mice, and 79% CD8^+^ and 20.9% CD4^+^ cells in cultures from Bonzo mice. We then investigated the behavior and effector function of primed CTLs against DBA/2 islets in vitro. We therefore imaged the infiltration of fluorescent CD8^+^ CTLs (CD8-τGFP, green) into the islets and their migration pattern within the islets by confocal microscopy (Figure 1A and Movie S1).

We also quantified several characteristic parameters of CTL migration in order to generate a dataset with which we could compare the data obtained in vivo (Figure 1B and Section 2.4). Immediately after plating the CTLs on the DBA/2-derived islets, we found a velocity of 5.39 µm/min (median, *n* = 54) and a track length of 160.04 µm (median, *n* = 54). The values for displacement, which describes the linear distance between the starting point and the end point of a track, and for the straightness, which shows the ratio of the track length/displacement, were 74.64 µm and 0.45, respectively (median, *n* = 54). The infiltration behavior of WT CTLs and their quantitative migration parameters (velocity: 5.42 µm/min; track length: 151.79 µm; displacement: 72.23 µm; straightness: 0.49; median; *n* = 56) were undistinguishable from CD8-τGFP CTLs (Figure 1A,B and Movie S2).

Because islets of Langerhans respond to altered blood glucose levels by changing their electrical activity and Ca^2+^ signals to release glucagon, insulin, and somatostatin, we infected islet cells with a Ca^2+^-sensitive GCaMP3 construct and measured Ca^2+^ levels before and during a T cell attack. Before T cell addition, resting islet cells showed spontaneous, intracellular calcium oscillations prior to the addition of T cells (Figure S2). Upon addition of CTLs, the oscillation frequency (and the basal signal) increased markedly (Figure S2). We observed death of multiple islet cells by apoptosis after the CTL attack, indicated by plasma membrane blebbing (Figure 1C and Movie S3). In Figure 1D, we visualized the polarization of cytotoxic granules (CG, red) towards the immunological synapse (IS) that was formed after contact with an islet cell by using CTLs from syb2-mRFP knock-in (SybKI) mice, in which the CG were labeled endogenously by red fluorescence. Immediately after CTL contact, the islet cell responded by a specific increase in intracellular calcium (Figure 1D, white arrows, Movie S4). These data demonstrate not only that CTL kill grafted islet cells in vitro, but also show the dynamics of the T cell attack with single granule resolution. We therefore continued to investigate CTL effector function in a living animal with the anterior eye chamber model.

### 2.2. Immune Cell Infiltration during Allorejection in the Anterior Eye Chamber Animal Model

**Figure 2:**
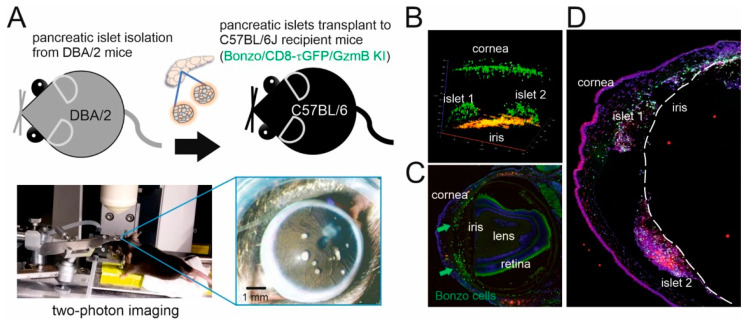
Immune cell infiltration during allorejection in the anterior chamber of the eye (ACE). (**A**) Schematic workflow of the ACE model. Pancreatic islets were isolated from DBA/2 donor mice and cultured for two days before transplantation into C57BL/6J recipient mice. Allorejection was followed by two-photon microscopy in the ACE. (**B**) Two-photon image of infiltrating GFP^+^ cells (green) of a Bonzo mouse at POD20. Cornea, iris, and islets are labeled. The image was acquired with z-stacks 560 µm thick with 4 µm interval distance. (**C**) Overview of a histological eye section with infiltrated immune cells (green) in Bonzo mice at POD7. Arrows point to infiltrated GFP^+^ cells. (**D**) Confocal overview of different subsets of immune cells infiltrating the islets at POD12 (CD3^+^ cells (magenta), CD14^+^ monocytes (red) and GFP^+^ cells (green)) in Bonzo recipient mice.

To investigate the effector function of CTLs against allograft tissue in vivo, we transplanted islets of Langerhans from donor DBA/2 mice into a C57BL/6 background recipient Bonzo mouse with fluorescently labeled T cells (Figure 2A). We followed the rejection course to observe the immune response, especially T cell infiltration dynamics, in the ACE by two-photon live imaging (Figure 2B) or histological staining to mark different immune cell subsets (Figure 2D). We started from observing general T cell infiltration in the ACE by using Bonzo recipient mice, in which the Cxcr6 gene is replaced by green fluorescent protein (GFP). Flow cytometry analysis of naïve splenocytes from Bonzo mice showed that 90% of GFP^+^ cells are CD8^+^ CTLs, while 6% are CD4^+^ T helper cells and the remaining 4% of GFP^+^ cells are not lymphocytes [14]. We followed infiltration of GFP^+^ cells in the ACE 7 and 12 days after transplantation (post-operational day (POD) 7 and POD12, respectively). Our histology data showed a clear structure of an eye with infiltrated GFP^+^ cells (green) in the ACE at POD7 (Figure 2C). We further characterized immune cell subsets on the islets after day 12 of implantation by staining with lymphocytes marker anti-CD3 (magenta) and monocyte marker anti-CD14 (red) (Figure 2D). We observed a high infiltration of immune cells on the islets, emphasizing the strength of in vivo models to obtain more comprehensive information of the immune response to allorejection under physiological conditions.

### 2.3. Infiltration of T Lymphocytes on Pancreatic Islets during Allograft Rejection

**Figure 3:**
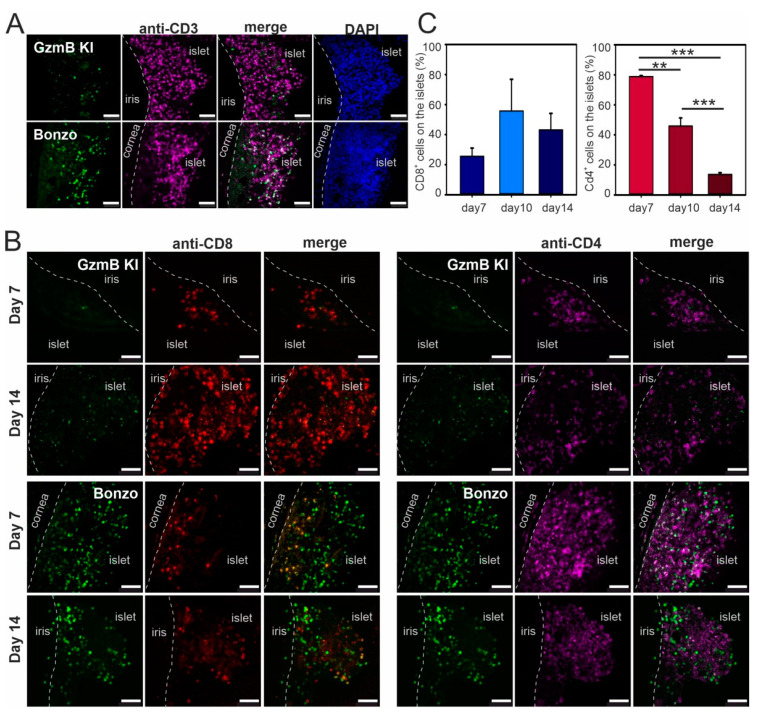
Infiltration kinetics of T lymphocytes into islets of Langerhans during allorejection. (**A**) Confocal images of histological sections of the eye containing pancreatic islets. Infiltrated lymphocytes were stained with anti-CD3 antibody (magenta) in GzmB KI (green, upper panel) and Bonzo (green, lower panel) mouse lines. The animals were sacrificed at POD14 for histological analysis. Scale bars: 50 µm. (**B**) Confocal images of histological eye sections containing pancreatic islets. Infiltrated T lymphocytes were further stained with anti-CD4 (magenta) and anti-CD8 antibodies (red) in GzmB KI (green, upper panel) and Bonzo (green, lower panel) mouse lines. The animals were sacrificed at POD7 and POD14 for histological analysis. Maximum intensity projection images are shown. Scale bars: 50 µm. (**C**) Analysis of T cell infiltration kinetics of CD8^+^ CTLs and CD4^+^ T helper cells during allorejection in Bonzo mice at POD7, POD10 and POD14. ** *p <* 0.01; *** *p <* 0.001. The statistical test used was the Mann-Whitney Rank Sum Test.

As T cell-mediated allograft rejection is precisely regulated over its time course, we next investigated the dynamics of T helper cell and CTL infiltration during rejection in ACE of Bonzo and GzmB KI recipient mice. We performed histology analysis following the rejection course and stained 14 µm thick eye sections with a lymphocyte-specific anti-CD3 antibody (magenta) to analyze lymphocyte infiltration on the islet at POD14 (Figure 3A). We observed a massive infiltration of T lymphocytes on the islets following the onset (~POD5–10, when T cells start to infiltrate into the ACE) to the peak of the rejection (~POD14–21, when many T cells infiltrate the islets and the islet starts to deform from the rigid edge). We further characterized T cell subsets from these infiltrating cells at the early phase of rejection (POD7) and the peak of rejection (POD14) as T cell-mediated immune rejection is highly regulated. We quantified the percentage of CD4^+^ T helper cells and CD8^+^ CTLs in the CD3^+^ lymphocyte population by staining the sequential section with an anti-CD4 antibody (magenta) and an anti-CD8 antibody (red) on the same islets during the course of rejection. Interestingly, our data showed that more infiltrating CD4^+^ T helper cells than CD8^+^ CTLs could be observed at the early rejection phase (POD7). In contrast, at the peak of the rejection (POD14) with a massive CD3^+^ lymphocytes infiltration, a strong increase of the CD8^+^ CTL population was observed in both Bonzo and GzmB KI mouse lines (Figure 3B). Quantitative analysis in Bonzo recipient mice revealed that the percentage of infiltrating CD8^+^ CTLs increases from POD7 (25.5 ± 5.5) to POD10 (55.8 ± 21.1) and POD14 (43.1 ± 11.0), while the percentage of infiltrating CD4^+^ T helper cells significantly decreases (POD7: 78.8 ± 0.6; POD10: 45.9 ± 5.4; POD14: 13.7 ± 1.1; Figure 3C). Thus, we conclude that CD4^+^ T helper cells arrive early in the course of rejection and CD8^+^ CTLs arrive later in the rejection phase, along with massive lymphocyte infiltration.

### 2.4. Visualization of Infiltrated Cytotoxic T Lymphocytes on Pancreatic Islets In Vivo

**Figure 4:**
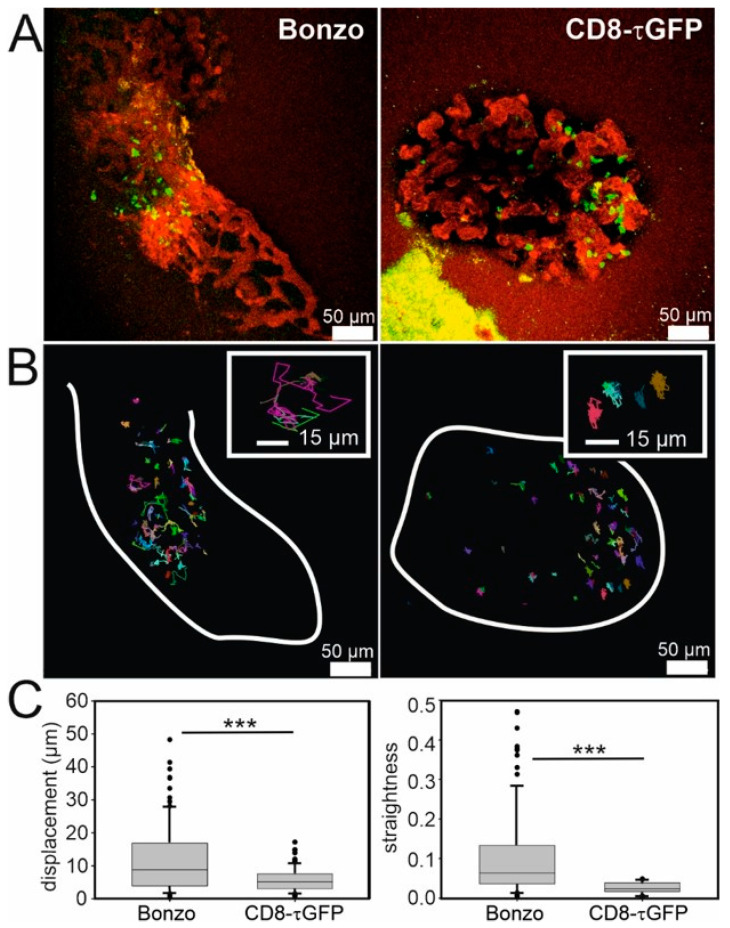
In vivo cell tracking of fluorescently marked lymphocytes rejecting allogenic grafts in the ACE at POD13. (**A**) DBA/2 islets of Langerhans (outlined by stained blood vessels in red) transplanted into the ACE of Bonzo (left) and CD8-τGFP recipient mice. Green dots represent GFP expressing cells. (**B**) Migration tracks of green cells from (A) over >15 min. White lines indicate outline of the islets. Insets are representative, zoomed sections of the islet. Note the difference of migration patterns. (**C**) Quantitative analyses of displacement (linear distance between a starting and an endpoint of a track, left) and straightness (ratio between displacement and track length, right) from (B). *** *p <* 0.001. The statistical test used was the Mann-Whitney Rank Sum Test.

We next wanted to focus on the CTL effector function against allograft tissue. Therefore, Bonzo and CD8-τGFP recipient mice were used to investigate the CD8^+^ CTL migration pattern on the islets. We imaged T cell infiltration into the islet and compared cell migration patterns of all T cell subpopulations in Bonzo recipient mice and of CTLs in CD8-τGFP recipient mice in vivo by two-photon microscopy (Figure 4). We observed that cell infiltration started from the cornea and that the onset of the rejection started after islet capillarization which usually occurred at POD4–5 (Figure 4A).

We next tracked all fluorescently labelled cells in both Bonzo and CD8-τGFP recipient mice that were visible for at least 15 minutes of our recording at POD13 (Figure 4B). From these tracking data, it is evident that GFP^+^ cells in Bonzo recipient mice had a much higher mobility than GFP^+^ cells in CD8-τGFP recipient mice. Quantitative analyses of displacement (linear distance between the starting point and the end point of a track) and straightness (ratio of the track length/displacement) revealed a statistically significant difference between Bonzo- and CD8-τGFP-mice for both parameters (Figure 4C; Displacement: Bonzo *n* = 101, median = 8.74 µm; CD8-τGFP *n* = 89, median = 5.09 µm, *p* < 0.001; straightness: Bonzo *n* = 101, median = 0.126, CD8-τGFP *n* = 89, median = 0.047, *p* < 0.001; Mann–Whitney Rank Sum Test). Comparison of these values with those values obtained from in vitro data (Figure 1B) revealed a ~10-fold reduction in both displacement (from 74.64 µm to 5.09 µm) and straightness (from 0.45 to 0.047). As cell velocity and mobility correlates with T cell activation, these data not only validate the effector status of CTLs against allograft islets in vivo, but also demonstrate the importance of obtaining data in vivo, where CTLs can communicate with other immune cells, rather than in vitro, where only CTL/target cell signaling is measured.

## 3. Discussion

We established the ACE model to study immune response underlying CTL effector function during allorejection. We demonstrated alloreactive T cell migration and their attack on islets of Langerhans in vitro by confocal microscopy. To account for the complexity of the immune system response to allografts, we further investigated the activity of CTLs in vivo on the transplanted islet in the ACE by two-photon microscopy. There are several observations from our imaging data demonstrating the efficient activation of alloreactive CTLs on the allograft islets. First, we visualized the apoptotic death of islet cells (Figure 1C and Movie S3) and a sudden disappearance of single islet cells after a sudden increase in intracellular calcium occurring upon contact by a single CTL (Figure 1D and Movie S4). Additionally, islets showed an immediate, dramatic increase in intracellular calcium concentration when co-cultured with CTLs (Figure S2). Second, we observed a rather stationary behavior of activated CD8^+^ CTLs (CD8-τGFP mouse) on islets compared to the mixed GFP^+^ T cell population, including subsets of other immune cells (Bonzo mouse) which showed a heterogenous migration velocity on islets in vivo (Figure 4).

While the death of islet cells is an unambiguous readout for CTL cytotoxicity, the migration velocity is an indicator for T cell activation and essential for subsequent T cell responses. It allows T cells not only to detect cognate antigen on the surface of APCs, but also to interact with other cells involved in the immune response, like CD4^+^ T helper cells and macrophages [15]. There are many reports stating that low T cell mobility correlates with the activation stage of the cells, mostly because of TCR recognition and synapse formation which also translates to effector competence [11,16,17]. Friedman et al. reported an altered behavior of CD8^+^ CTLs during progression of autoimmune attack that in the early stage of T cell infiltration into the islet, T cells interact with APCs through TCR recognition, which results in stable contacts. Additionally, T cell-APC encounters support production of interferon-γ (IFN-γ) by effector T cells at this stage and the killing of islet cells also occurs. At a later stage of infiltration, T cell mobility increased and the cytokine production was lost, despite a higher number of infiltrating APCs that were able to trigger T cell signaling in vitro [16].

Concerning the complexity of adaptive immune system responses against allografts, we made the interesting observation that the temporal infiltration pattern of T cells during rejection was highly ordered, with an enrichment of CD4^+^ T helper cells on the islets long before the arrival of CD8^+^ CTLs (Figure 4). It has been shown that there are on average 810 pre-existing DCs per islet of Langerhans which constitutively present β cell-derived peptides bound to MHC class II molecules on their surface [18]. Additionally, a small fraction (10%) of islet cells also expresses MHC II on their surface [19]. These factors might explain the early arrival of CD4^+^ T helper cells due to the allorecognition in the eye. Furthermore, while immune responses during allorejection are highly regulated, direct and indirect allorecognition represent distinct mechanisms involving different APCs, T cells, and antigen determinants. T cells activated directly and indirectly could be either reinforcing or suppressing each other, which influences the survival of allografts. It is possible that in recipients of MHC class I allografts, CD4^+^ T helper cells are activated exclusively through indirect allorecognition that secrete cytokines like IL-2 and IFN-γ for the direct activation of other CD4^+^ T helper cells or the differentiation of CD8^+^ CTLs to recognize donor MHC class I peptides in a direct manner. This might explain our observation that the kinetics of CD4^+^ T helper cells and CD8^+^ CTLs arrival on allograft islets appear in a sequential manner (Figure 3). In addition, early inflammatory direct alloresponses associated with IFN-γ and tumor necrosis factor alpha (TNF-α) cytokine production and that subsequently induce donor MHC class II expression on endothelial cells presumably enhance allo-MHC antigen processing by recipient APCs and result in indirect activation of T cells. Therefore, the alloresponses act synergistically between cells to reject the allografts [6].

In principle, confocal and two-photon imaging is possible in almost every location of a living mouse. Regarding the study of immune responses, the most popular areas have been spleen, lymph nodes, brain and the dorsal skinfold chamber [16,17,20,21,22,23]. While all these models delivered important information on immune cell function in vivo, the ACE model has obvious advantages over these systems with regard to microscopic imaging. Because the eye itself is an optical apparatus, it allows undisturbed imaging with unparalleled penetration depth and resolution without the need to clarify the tissue. Therefore, non-invasive longitudinal live imaging of single immune cells as well as in-depth analysis of their mobility and interaction with other cells is easily possible. Furthermore, it appears likely that super-resolution imaging techniques like stimulated emission depletion (STED) microscopy [24] will in the future even allow the detection of individual cytotoxic granules. The availability of the mouse lines reported here to specifically label CTL populations (CD8-τGFP mouse) and single CGs (GzmB KI and SybKI) will prove very helpful to not only understand the mechanism and regulation of CTL-mediated killing in vivo, but also to investigate the killing in gene-deficient mice that resemble severe human immune diseases.

## 4. Materials and Methods

### 4.1. Mice

DBA/2 mice (12–30 weeks old) were purchased from Jackson Laboratory (Bar Harbor, ME, USA; #000671) and used as donor mice for islet of Langerhans isolation. The recipient mouse lines used in the study were all with C57BL/6 background strain. GzmB-mTFP KI mice were generated by clustered regularly interspaced short palindromic repeats (CRISPR)-CRISPR-associated protein 9 (Cas9) technology to have GzmB fused to monomeric teal fluorescent protein (mTFP) under the control of the endogenous promoter. A detailed description of the mice will be published elsewhere. Bonzo mice were purchased from Jackson Laboratory (#005693). The CD8-τGFP mouse was generated by crossing a previously published τGFP line [25] with a CD8a-Cre line (Jackson Laboratory, #008766). All animal experiments were performed according to German law and European directives, and with permission of the state of Saarland (Landesamt für Gesundheit und Verbraucherschutz; animal license number 41-2016; approval date: November 8, 2016).

### 4.2. Plasmids and Antibodies

An adenovirus packed GCaMP3 construct was used to infect islet cells. The following antibodies were used for immuno-staining: Alexa Fluor 647 Rat anti-Mouse CD3 Molecular Complex (Clone 17A2, BD Pharmingen, Heidelberg, Germany), anti-Mouse CD4-APC (clone GK 1.5, Life Technologies, Carlsbad, CA, USA), anti-Mouse CD8a-BV421 (clone 53-6.7, Biolegend, San Diego, CA, USA), recombinant anti-CD14 (ab133335, Abcam, Cambridge, UK).

### 4.3. Cell Culture

DBA/2-specific T cells were obtained from recipient mouse lines (WT, GzmB KI, Bonzo or CD8-τGFP). Briefly, splenocytes were isolated from 8 to 20 week-old mice, as described before [26]. Splenocytes were incubated with crude DBA/2 lysate (~1.5 mg/mL total protein concentration) in RMPI culture medium containing 50 µM β-mercaptoethanol, 10% fetal calf serum, 1% Penicillin/Streptomycin and supplemented with 50 U/mL IL-2 for two days. Blast cells were generated after two days in culture. 100 U/mL IL-2 was added to the culture medium at day 3 to induce proliferation of T cells. T cells were separated from mixed splenocytes at day 3 or 4 using Lymphocyte Separation Medium 1077 (PromoCell, Heidelberg, Germany) with a gradient centrifugation at 400× *g*, 30 min at room temperature. For T cell killing and migration experiments, day 4 and 5 effector T cells were used for co-culture with islet cells after lymphocyte purification.

### 4.4. Isolation of Islets of Langerhans

Islets of Langerhans were isolated as described previously [27]. Briefly, mice were killed by cervical dislocation. The pancreas was inflated through the common bile duct with 3 mL of a Ringer solution containing 50 µg Liberase TL (Merck, Darmstadt, Germany). After injection, the pancreas was removed from the mouse abdomen and digested at 37 °C in 5 mL of Ringer solution with gentle agitation for 12 min in a water bath. The digestion was stopped by adding 25 mL of cold (4 °C) Ringer solution. The pancreas then was smashed mechanically. Undigested debris was separated from islets and exocrine material by filtration through a steel mesh (200 µm mesh size). Finally, islets were separated from exocrine material by density centrifugation (900× *g*, 18 min) using a Ringer/Histopaque gradient (Histopaque 1077, Merck). Islets were collected from the interphase, while exocrine material remained in the pellet. The exocrine material was later used for intraperitoneal injection into mice after islet implantation and T cell culture as DBA/2 antigen for T cell priming. After isolation, islets were collected by filtering the supernatant through a 70 µm strainer, washed and resuspended in RPMI 1740 cell culture medium containing 10% FCS and 10 mM HEPES and kept in an incubator (37°C, 5% CO_2_) for up to 4 days before transplantation.

### 4.5. Transplantation of Islets of Langerhans into the Anterior Chamber of the Eye

Transplantation was performed as described previously [28]. Briefly, mice (12–20 weeks old) were anesthetized with isoflurane. An opening (500 µm) was cut on the cornea with a sharp injection needle (30 G). A blunt cannula (0.4 mm) preloaded with 10–20 islets was inserted through this opening. The cannula was connected to a foot panel-steered injector through which the islets were injected into the eye under optical control (binocular microscope). After transplantation, mice were kept for 4 days under pain relief medication (Caprofen, 5 mg/kg bodyweight) before any further experimental manipulation.

### 4.6. Confocal Imaging

T cell behavior was recorded on/with islet cells in vitro by confocal microscopy (LSM 780). The live imaging was performed at 37 °C. The image acquisition information is stated in each figure, including z-stack and time resolution. For live cell imaging, isolated islets were cultured in an 8-well glass chamber (Sarstedt, Lot 9022111) for 8–10 days until the islets are stably adhered in the well. 1–2 × 10^5^ effector T cells were added to islets per well to record T cell migration and killing on islet cells. WT T cells were loaded with 500 ng/µL calcein-AM (Life Technologies) for 15 min at room temperature (RT) before imaging. The histological eye sections were also imaged by confocal microscopy. The images were acquired with a 40× Plan-Apochromat objective (NA 1.3). The GzmB KI and Bonzo fluorescence were excited at 488 nm excitation wavelength, anti-CD8a BV421 was excited at 405 nm, and anti-CD3-Alexa647 and anti-CD4-APC were excited at 647 nm wavelength. The image stacks were acquired with 10 µm total thickness at 1 µm interval distance. The maximum intensity projection images are shown in live cell imaging data and in histology data.

### 4.7. In Vivo Imaging by Two-Photon Microscopy

Transplanted mice were pre-anesthetized with isoflurane in a box. Animals were then mounted to a 3-point head holder with a respirator mask for continuing anesthesia. The mouse was placed on a heating plate and the head was tilted with the corneal surface of the transplanted eye in perpendicular orientation to an objective of an upright microscope. A two-photon microscope (LSM880, Carl Zeiss Jena, Jena, Germany) was used for live imaging. The system was equipped with a 20× Plan-Apochromat water immersion objective (Carl Zeiss Jena, NA 1.0). The data were acquired as time stacks (4D) and are displayed as maximum intensity projections. The acquisition of stack thickness and time were different depending on the size of the islet and on the number of infiltrating cells, therefore the acquisition information is indicated in each dataset. For data acquisition, Zen software (Zeiss, Jena, Germany) was used. Data analyses and three-dimensional (3D) reconstructions were performed by Imaris9.3 (Bitplane AG, Zurich, Switzerland). Deconvolution was done with Autoquant 3X (Media Cybernetics Inc., Rockville, MD, USA).

### 4.8. Histology and Immunohistochemistry

For immunohistochemistry, mice were deeply anesthetized by an intraperitoneal injection of a mixture of ketamine (280 mg/kg bodyweight) and xylazine (20 mg/kg body weight). The mice were intracardially perfused with phosphate-buffered saline for 5 min followed by a fixative containing 4% para-formaldehyde for another 5 min. After perfusion, the transplanted eye was isolated and post-fixed in the same fixative for another 4 h. After washing once with PBS, the eye was incubated in 30% sucrose solution overnight (4 °C) before embedding in Tissue-Tek O.C.T. (Sakura Finetek Europe B.V., Staufen, Germany). For embedding, the eye was snap-frozen surrounded by Tissue-Tek O.C.T. for 1 min into frozen 2-methylbutane at –80 °C. The eye samples were then cut into 14 µm thick sections by a cryostat (Leica 3050S) at –16 °C. Slices were collected on Superfrost+ slides (VWR) and stored at –80 °C for further immunohistochemistry.

For immunostaining, eye sections were air-dried for 30 min at room temperature from –80 °C storage. Eye samples were washed twice with PBS, permeabilized with 0.1% triton (permeabilization buffer) for 10 min, followed by blocking with extra 2% bovine serum albumin in permeabilization buffer for 30 min at RT. Afterwards, samples were incubated overnight at 4 °C with anti-CD3-Alexa647 (1:200), anti-CD4-APC (1:200), and anti-CD8-BV (1:200). Samples were mounted with fluorescence mounting medium (Dako, Agilent, Santa Clara, CA, USA) after washing several times with PBS and stained with DAPI (1:1000).

### 4.9. Statistics and Image Analyses

Statistical differences in data were calculated with the Mann–Whitney Rank Sum Test. Data were analyzed with ImageJ v1.46 [29], Excel (part of Office 2013, Microsoft), SigmaPlot 13 (Systat Software Inc., Erkrath, Germany) and Imaris 9.3 (Bitplane AG) and graphed using Affinity Designer Software (Serif Ltd, Nottingham, UK).

## Supplementary Materials

Supplementary materials can be found at https://www.mdpi.com/1422-0067/21/13/4660/s1. Figure S1: DBA/2-specific CTL generation. T cells were generated from splenocytes and incubated two days with exocrine material from DBA/2 islet preparation. (A) Confocal images of T cells cultured for 5 days after lymphocytes gradient separation and stained with anti-CD8-BV (blue) and anti-CD4-APC (magenta) isolated from WT (left panel), GzmB KI (middle panel) and Bonzo mice (right panel). (B) Analysis of CD8^+^ and CD4^+^ cell populations in three different mouse lines after 5 days in culture (from A). Figure S2: Intracellular calcium response in islet cells upon T cell attack. (A) Confocal images show islet cells infected with AV-GCaMP3 construct (green) before and after SybKI CTLs (red) co-cultured. Maximum intensity projection images are shown. (B) Analysis of GCaMP3 signal from live cell imaging recording. GCaMP3 signals from six individual islet cells are shown as representatives from 83 measured cells. Images were recorded for 6 min before T cell application and 76 min after adding T cells. The images were acquired in 5 µm slices with 1 µm interval distance and ~0.1 Hz acquisition frequency. Movie S1: Confocal live recording of calcein-loaded WT CTLs (green) infiltrating a pancreatic islet. Movie S2: Confocal live recording of CD8-*τ*GFP CTLs (green) infiltrating a pancreatic islet. Movie S3: Confocal live recording of WT CTLs co-cultured with islet cells infected with AV-GCaMP3 (green). Cell blebbing indicative of apoptosis can be seen in two islet cells. Movie S4: Confocal live recording of SybKI CTLs (red) co-cultured with islet cells which were infected with AV-GCaMP3 (green).

## Abbreviations

CTL	cytotoxic T lymphocytes
TCR	T cell receptor
MHC I	major histocompatibility class I complexes
IS	immunological synapse
CG	cytotoxic granules
GzmB	granzyme B
DCs	dendritic cells
APC	Antigen-presenting cells
ACE	anterior chamber of the eye
GzmB KI	GzmB-mTFP knock-in mouse
POD	post-operational day
