# Supplementary material for: Investigation of Cytotoxic T Lymphocyte Function during Allorejection in the Anterior Chamber of the Eye
Source: Int J Mol Sci. 2020 Jun 30;21(13):4660. doi: 10.3390/ijms21134660 (PMC7369940; doi:10.3390/ijms21134660)
Supplement: Supplementary file 1 [file ijms-21-04660-s001.zip › ijms-825492 suppl final/ijms-825492 suppl.pdf]

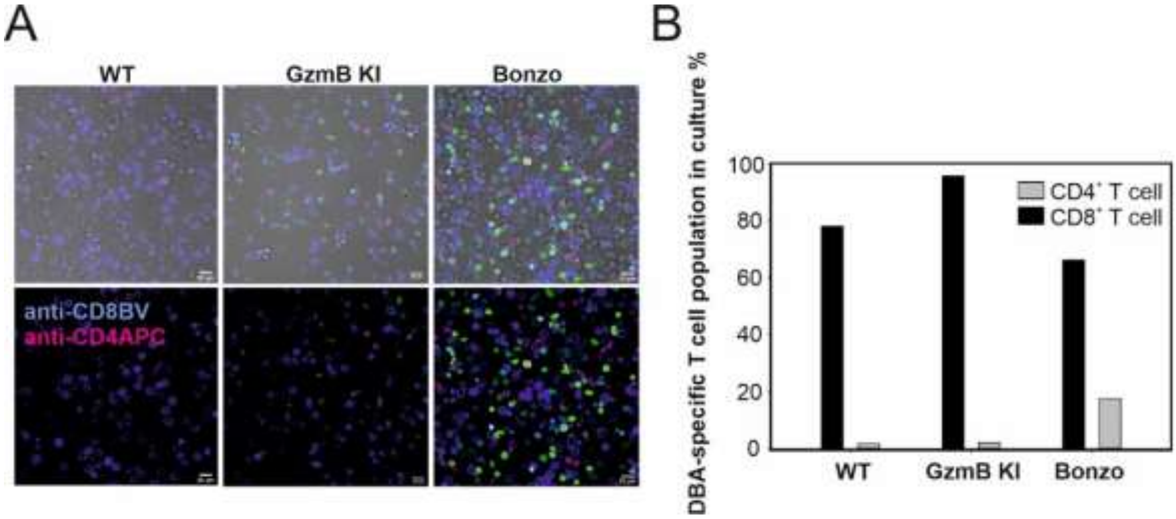

Figure S1. DBA/2-specific CTL generation. T cells were generated from splenocytes and incubated two days with exocrine material from DBA/2 islet preparation. (**A**) Confocal images of T cells cultured for 5 days after lymphocytes gradient separation and stained with anti-CD8-BV (blue) and anti-CD4-APC (magenta) isolated from WT (left panel), GzmB KI (middle panel), and Bonzo mice (right panel). (**B**) Analysis of $CD8^+$ and $CD4^+$ cell populations in three different mouse lines after 5 days in culture (from A).

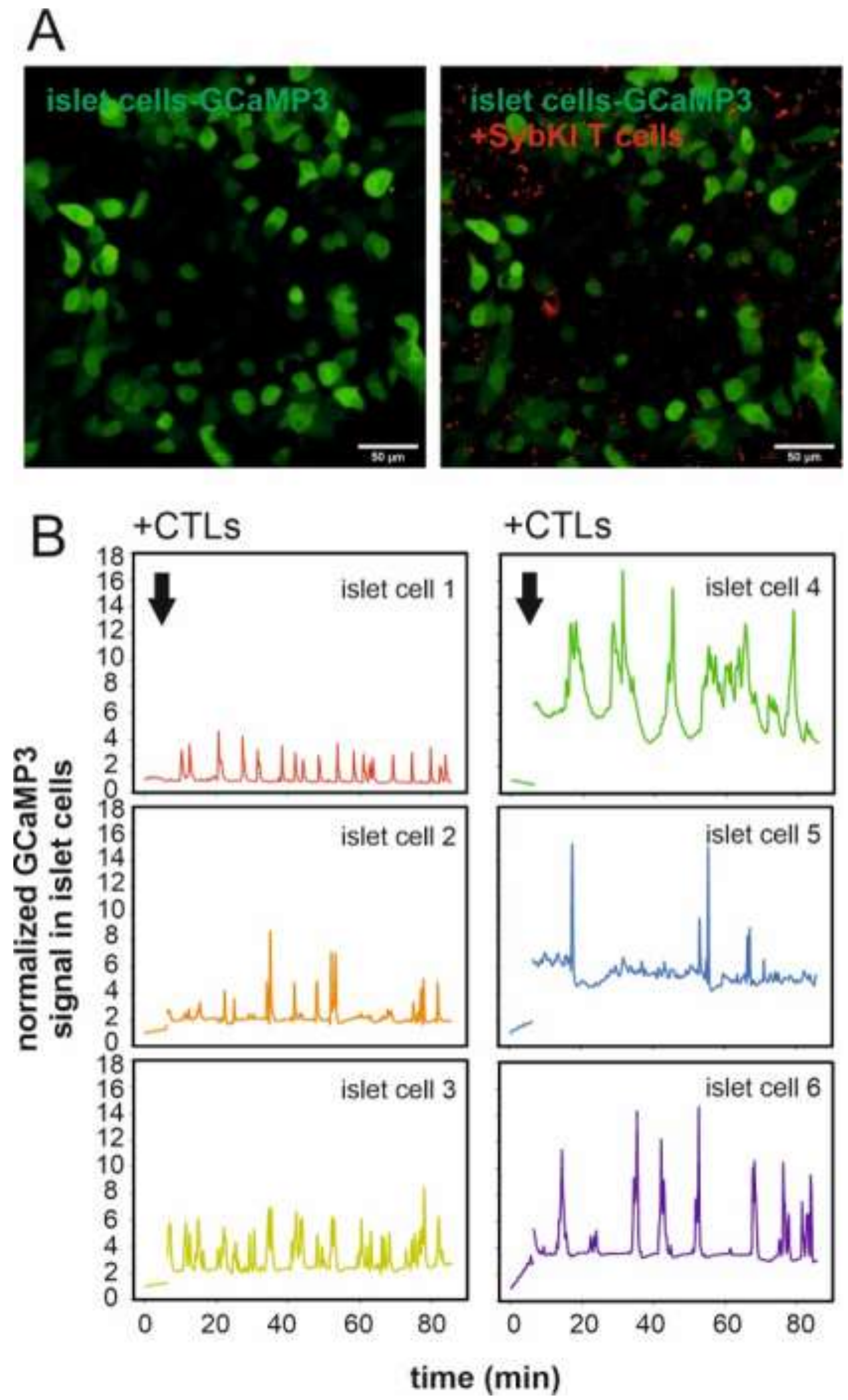

Figure S2. Intracellular calcium response in islet cells upon T cell attack. (**A**) Confocal images show islet cells infected with AV-GCaMP3 construct (green) before and after SybKI CTLs (red) co-cultured. Maximum intensity projection images are shown. (**B**) Analysis of GCaMP3 signal from live cell imaging recording. GCaMP3 signals from six individual islet cells are shown as representatives from 83 measured cells. Images were recorded for 6 min before T cell application and 76 min after adding T cells. The images were acquired in 5 µm slices with 1 µm interval distance and ~0.1 Hz acquisition frequency.
